# Establishing accountability and promoting rights: the WHO QualityRights contribution to mental health, recovery and community inclusion

**DOI:** 10.1192/bjo.2025.10835

**Published:** 2025-09-12

**Authors:** Julian Eaton

**Affiliations:** Professor in Global Mental Health, Department of International Public Health, Liverpool School of Tropical Medicine, Liverpool, UK

**Keywords:** Human rights, mental health services, quality improvement, global mental health, patients and service users

## Abstract

Attention to human rights as a central pillar of global mental health work has shifted from a focus on the right to healthcare to a deeper examination of the quality of care received, and to the way in which people with mental health conditions are treated in all aspects of life. The QualityRights programme is the World Health Organization’s flagship guidance for promotion of rights-based approaches to mental healthcare, and a means of holding service providers to account for quality of care provided. A recent evaluation of the QualityRights e-training package demonstrates promising impact on attitude change of participants, raising the prospect of an efficient scale-up of efforts to improve dignity in services and reduce stigma and discrimination.

**Figure:**
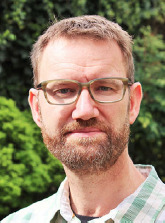

## Global mental health and human rights

Attention to the human rights of people with mental health conditions has long been a stated priority for the field of global mental health. This was the conclusion of the important 2007 Lancet Global Mental Health series, written by a diverse range of global leaders in the field as it emerged in its current form: ‘… we call for action to scale up coverage of services for mental disorders, and to strengthen protection of the human rights of those with mental disorders’.^1^

Subsequent research and implementation has primarily focused on reducing the huge mental health treatment gap, which was described as a ‘failure of humanity’.^2^ A similar moral and rights-based argument was used to make the case for increased investment in scaling up of mental health interventions globally – for example, by Patel and Farmer.^3^ This clear moral basis brought a sense of urgency and common endeavour, deliberately drawing on principles and methods of global public health to respond to defined research priorities.^4^ Human rights were particularly central to the agenda of systems reform, with long-stay institutions seen as the most egregious examples of human rights abuses, and innovation to decentralise and integrate services into general healthcare as the means of increasing equitable coverage or, in other words, the right to health.^5^ Other key approaches, such as task sharing to address the lack of specialist mental health personnel, and provision of scalable psychological interventions to complement biomedical approaches, are also underpinned by considerations of equity of access and promoting agency. More recently, digital solutions have been promoted on the basis of increasing access to care in exciting ways – although there are importance concerns about the digital divide.

## The WHO QualityRights programme

Aside from the ‘right to health’ case for scaling services, the direct human rights component of this call to action has been less prominent, but is an essential complement to any meaningful services reform. The World Health Organization (WHO) QualityRights programme, launched in 2012, has been instrumental in bringing these two areas together. The linking of quality of services with human rights addresses an important critique of the field – that global mental health risks simply exporting the far from satisfactory service models developed in high-income countries. The bar for attainment of greater service coverage must include a quality component because evidence suggests that, even among the services that do exist, less than a quarter currently meet minimally acceptable quality standards.^6^

In addition to providing resources and guidance about rights-based principles and approaches, QualityRights provides tools for evaluation of services (including by people who use them) – an important accountability mechanism, the absence of which has arguably allowed the persistence of unacceptable services for far too long. One way of making task sharing more consistent is by clearly defining the roles and associated competencies of different actors. When this is done, there is no evidence that standards are reduced.^7^ Evaluation of quality is an extension of promoting human rights in QualityRights, and consistent and transparent evaluation of quality in services, if linked to accountability mechanisms, can carry along public and user confidence during processes of service reform.

Users of services have had an important influence in championing human rights approaches to mental health. While remaining diverse, there has been a deliberate alignment towards the values and priorities of the wider disability movement, defined largely by the Convention on the Rights of Persons with Disabilities.^8^ Importantly, QualityRights uses the Convention as a framework for establishing principles and setting standards, increasing its credibility. In a major trial of QualityRights in Gujurat, India, this also meant recognising the role of peer support in challenging some of the more ingrained attitudes that have robbed service users of dignity.^9^

A full realisation of human rights-based approaches to delivering services will certainly be challenging, but the practical tools needed to realise this are now emerging.^10^ It holds the promise not only of a better experience for those using services, but also of improved outcomes and a more trusting therapeutic relationship between clinicians and patients. A good example is the emphasis on consensual approach to engagement with patients in the WHO mhGAP Intervention Guide. This is of value in everyday practice, but also to set ethical standards that will protect the profession’s reputation in the context of widespread use of legally sanctioned coercive practices,^11^ and co-option for political ends, from the old Soviet Union to current-day Iran.^12^ There remain significant tensions to be navigated in relation to the United Nations’ Convention on the Rights of Persons with Disabilities Committee’s position of total abolition of non-consensual treatment (reinforced in a General Comment on Article 12).^13^ Few countries have aligned to this, with many explicitly defending a position contradicting the General Comment position on containment, guardianship and treatment choice.^14^

Building on the initial focus in global mental health around the right to health, the movement of people with lived experience has made the case for realisation of the full range of rights that people should expect: community and family life, economic and cultural rights, political participation and others that have been routinely denied people with mental health conditions. A shift to community-based, comprehensive, person-centred approaches in service design demonstrates some adoption of this principle. These services tend to increase access and promote more holistic treatments, improving clinical outcomes, functioning and quality of life, including for carer-givers.^15^ Widespread ratification of the Convention by countries globally establishes an important mechanism for holding service providers to account, and QualityRights is one means to achieve this. Such a broad assertion of rights in all areas of life might be a means of realising rights within, but also beyond, services, but major challenges remain; stigma is pervasive among both the public and policy-makers, and service user groups remain relatively weak and poorly resourced, denying them opportunities for meaningful political engagement to hold service providers accountable.

## Increasing access to training in rights-based approaches

The evaluation of the QualityRights e-training published in this edition, described by Funk and colleagues in an article in this edition,^16^ saw substantial shifts in negative attitudes towards people with mental health conditions reported among trainees. There is widespread assertion of human rights as a foundational principle in mental health service provision, but consistent documentation of this is not being realised in reality.^17^ The prospect of tackling stigma and discrimination, those most pernicious of challenges to improving quality of life in the community, while providing more dignified and better-quality services, is potentially revolutionary. The fact that this was achieved through online delivery raises the prospect of a far more scalable means of changing attitudes and promoting less coercive, more rights-based mental health services with the urgency that this endeavour deserves.
